# Information sources as determinants of use of formal long-term care: a cross-sectional study in Taiwan

**DOI:** 10.1186/s12913-025-12814-6

**Published:** 2025-07-03

**Authors:** Yu-Hung Chang, Chia-Hui Hsu, Yu-Chun Tseng, Wan-Chun Yang, Hung-Yi Chiou

**Affiliations:** 1 Institute of Population Health Sciences, National Health Research Institutes, 35 Keyan Road, Zhunan Town, Miaoli County 350 Taiwan; 2 School of Public Health, College of Public Health, Taipei Medical University, Taipei, Taiwan

**Keywords:** Long-term care, Information sources, Migrant caregivers, Utilisation

## Abstract

**Background:**

An ageing population has heightened the need for long-term care (LTC) access. It is not well understood how information sources, both formal and informal, impact the utilisation of LTC. This study aims to investigate the influence of formal and informal sources on the utilisation of LTC services by Taiwanese families employing migrant live-in caregivers.

**Methods:**

This study employed a cross-sectional design and used a structured telephone survey to collect data from 441 registered employers from households employing migrant caregivers. This included 216 families using LTC and 225 not using LTC. We collected data on the characteristics of employers and care recipients, as well as LTC-related information sources. Our formal sources (FS) included government promotional events, LTC community site volunteers, healthcare providers, and local LTC management centres, while informal sources (IS) comprised community leaders, friends and relatives, TV, print media, internet, and patient support groups. We assessed perceived information gaps by asking questions about reasons for not using LTC. We used logistic regressions to analyse the associations of FS and IS with LTC use, employer and care recipient characteristics, and perceived information gaps.

**Results:**

A higher number of FS was positively associated with LTC usage, while an increase in IS was negatively associated. FS and IS were associated with employer socio-demographics such as gender, education level, and care recipient factors, including living arrangements and care needs. FS reduced perceived information gaps, but IS did not.

**Conclusions:**

Information sources significantly influence the use of LTC among families employing migrant caregivers. Our findings suggest that LTC authorities should implement strategies to enhance the accessibility of formal information sources, while also leveraging informal sources more effectively to support accurate and timely LTC decisions for these families and the wider public.

**Supplementary Information:**

The online version contains supplementary material available at 10.1186/s12913-025-12814-6.

## Introduction

As the world’s population ages, the demand for long-term care (LTC) services has consistently increased [1, 2]. By 2050, the proportion of older adults globally is expected to reach 16.7%, nearly doubling from 8.5% in 2015 [3]. From 2020 to 2040, global LTC needs are projected to grow by 47%, especially in Europe, North America, and East Asia [1]. Along with aging, the prevalence of disability contributes to the increased use of LTC services [4]. In OECD countries, rising severe disability rates expand the proportion of older adults requiring support [2]. As informal care from family caregivers become more limited, governments face growing pressure to ensure accessible and affordable LTC services [5].

In Taiwan, the older population surpassed 14% in 2018, will exceed 20% by 2025, and is projected to reach 30% by 2039, according to the National Development Council [6]. The disability rate among those aged 65 over is approximately 18%, based on a national survey by the Ministry of Health and Welfare (MOHW) [7], highlighting substantial LTC needs. As population ageing accelerates, the public increasingly expects the government to take an active role in LTC system development and reform [8].

In 2007, Taiwan implemented its first 10-year Long-Term Care plan (LTC 1.0), which provided national funding and introduced local care management [9]. However, challenges such as funding constraints, limited service flexibility, workforce shortage, and administrative inefficiencies prompted reform [8, 10]. In 2017, MOHW initiated the Long-term Care 2.0 Plan (LTC 2.0). LTC 2.0 introduced a three-tiered service system, the ABC network, to deliver integrated community-based care [9]. Tier A (Integrated Community Service Centre) coordinates LTC resources and develops care plans. Tier B (LTC Service Institution) provide specialised services such as home care, day care, and nutritional support. Tier C (Local Service Stations) offers neighbourhood walk-in services for older adults with mild disabilities, aiming to prevent further decline [10, 11].

The LTC 1.0 provided a variety of government-funded care including home care, day care, adult foster care, home nursing, rehabilitation, transportation, meal delivery, and respite services, through an in-kind benefit system [10, 12]. LTC 2.0 expanded service coverage from 8 to 17 categories, incorporating dementia care, small-scale multi-functional services, family support services, disability prevention and delay programmes, and transitional care services linking hospital discharge plans to community care [9, 10, 12].

By 2023, MOHW reported 80% coverage rate for individuals needing LTC services [13], leaving about one-fifth not using LTC 2.0 services. Among individuals in need of LTC, the majority relied on family members or paid caregivers, while few received formal care from residential institutions such as nursing home [7, 8, 14, 15]. These gaps in coverage and utilisation of LTC 2.0 services underscore ongoing policy challenges in meeting LTC needs.

In Taiwan’s LTC system, the widespread use of migrant live-in caregivers may contribute to the underutilisation of formal LTC services. Live-in caregivers are domestic workers who provide personal care services and perform some household tasks while living in care recipients’ home [16]. Since 1992, the government has permitted their employment under labour regulations to assist individuals with disabilities [15]. To hire one, employers must apply through the Ministry of Labour (MOL), first seeking local caregiver matching through local Long-Term Care Centres. If no match is found, they may apply to MOL for migrant caregiver approval [17].

Migrant live-in care workers provide 24-hour home care, sharing responsibilities with family members and complementing family care [18]. As paid caregivers without prior social ties with care recipients, their services are classified as formal care [15, 19]. They are preferred for their lower cost and longer working hours compared to local caregivers [8, 9], despite challenges such as language and cultural barriers [20]. Even after the implementation of the LTC 1.0/2.0 plan, hiring migrant care workers remains an alternative care model, as LTC services typically offer only limited daily hours, which may not meet intensive care needs [14, 18].

In 2023, 184,297 migrant live-in care workers were employed in Taiwan, according to statistics from MOL [21]. That same year, MOHW estimated that 860,398 individuals required LTC services [13]. Given a one-to-one caregiving ratio, 21.4% of those in need used migrant caregivers, but only 88,592 were covered by the LTC 2.0 plan [22], suggesting that over half did not access LTC 2.0 services. Therefore, migrant caregiving may substitute for, rather than complement, LTC 2.0. Bridging this gap is essential to achieving comprehensive coverage.

LTC service use is shaped by various factors [23–25], especially information accessibility and quality [26, 27]. In healthcare, information supports decisions to seek help, accept care, and managing post-care consequences [28]. This need for information supports individuals in coping with health-threatening situations, participating in decision-making, and initiating behavioural changes [29]. Such situations involve perceived risks of illness or care burden that are seen as likely and potentially serious, prompting individuals to seek preventive or supportive actions they consider effective and manageable in terms of time, effort, or cost [30]. Health information-seeking improves cognitive, behavioural, and health outcomes, along with increased patient satisfaction [31–33], while ethnic disparities in information access contribute to healthcare inequalities [34].

Bradley et al.’s framework [35], an extension of the Anderson behavioural model, identifies attitudes and knowledge, social norms, and perceived control as psychosocial mediators between need and enabling factors and actual LTC use. These psychosocial components, shaped by both individual and contextual influences, emphasise cognitive and normative pathways in decision-making. Notably, knowledge in this framework overlaps with information and includes content, sources, and accessibility, directly influencing service use through its interaction with attitudes in shaping individual decisions.

Individuals and their families often encounter challenges in accessing LTC services due to insufficient system information [36]. In response, they typically consult various sources, including formal (e.g., physicians, official institutions) and informal (e.g., family, friends, mass media, online platforms) [28, 37, 38]. The use of information sources is shaped by demographic and socioeconomic characteristics, as well as specific caregiving contexts [37–41], which in turn affect how individuals seek, access and apply health information.

Prior studies suggest that perceived credibility of formal (FS) and informal sources (IS) influences individuals’ decisions to utilise health and care services, particularly when the information is regarded as trustworthy [26, 42]. FS provide professional and credible information that facilitates informed decision [38, 43–45], while IS shape perceptions of needs and motivate behavioural changes and service use through social norms and trusted relationships [28, 46, 47]. For example, IS often guide early decisions (e.g., surgery choice), whereas FS gain importance during recovery [28]. When perceived as credible, both FS and IS can support information access and service uptake, through professional and institutional authority in the case of FS, and interpersonal trust in the case of IS [46].

In Taiwan, employing migrant live-in caregivers has become a common strategy for meeting LTC needs. Drawing on data from a nationwide survey of such households, this study examines how different information sources affect the utilisation of LTC 2.0 services. Grounded in prior theory and evidence, we propose and test the following hypotheses:

- Hypothesis 1: A greater number of FS and IS is associated with an increased likelihood of LTC service utilisation.
- Hypothesis 2: The characteristics of employers, care recipients, and household contexts (e.g., gender, education level, caregiving role, and care needs) are associated with the types of information sources accessed.
- Hypothesis 3: A greater number of FS and IS is associated with a lower likelihood of reporting perceived information gaps regarding LTC services.

## Methods

### Data

Our study utilised data from a structured telephone survey conducted in June 2022, targeting registered employers of migrant live-in caregivers in Taiwan. The sampling frame was derived from MOL registry, which includes individuals legally required to register when hiring a migrant caregiver. The survey used a standardised, close-ended questionnaire administered by trained interviewers to examine LTC 2.0 service utilisation, experiences, and satisfaction, focusing on differences between LTC users and non-users. No semi-structured or open-ended interview components were included. The English version of the questionnaire is available (see Additional file 1 “Survey Questionnaire”).

We anticipated 400 completed interviews, with equal numbers of LTC 2.0 users and non-users (*n* = 200 each). LTC utilisation status was confirmed in advance by cross-referencing official LTC 2.0 service records with the MOL registry, which also served as the sampling frame. A stratified random sampling approach was employed, allocating participants proportionally across four geographic regions (North, Central, South, and East) to form eight strata. Assuming a 10% completion rate [48], 4,000 employers were randomly selected across strata.

The telephone survey was conducted over two weeks using a computer-assisted telephone interviewing system. A pilot test was conducted to access the questionnaire and procedures. In the first round of contact, 3,082 calls yielded 367 completed interviews. A second round of 1,682 calls to previously unreachable cases added 101 more completed interviews. In total, 468 interviews were completed. Unsuccessful contacts were due to no answer, refusals, incorrect numbers, and ineligibility (e.g., no longer employing a migrant caregiver) (see Additional file 2). After excluding 25 non-registered employers and two misclassified users, the final analytical sample included valid 441 respondents: 228 users and 213 non-users.

### Variables

#### Use of LTC services

Participants were categorised as LTC service users and non-users based on the recent service records and further confirmed during interviews. Respondents were asked whether care recipients had used LTC services in the past three months. This recall period was selected to balance recall accuracy and the need to capture infrequent services like professional or respite care. A shorter window may miss such use, while a longer one risk recall bias.

#### Sources of information on LTC services

Respondents were asked to identify their primary sources of LTC information from 11 options, including government promotional events, community leaders (village/neighbourhood representatives), friends and relatives, TV, print media, internet, LTC sites volunteers, patient/family groups, LTC care workers, healthcare providers (medical care institutions), and LTC management centres. These options were derived from prior literature [37–39, 49–52], and refined by two LTC experts to reflect Taiwan’s LTC policy context. Sources were categorised as formal (FS) and informal (IS) sources. FS included government promotional events, healthcare providers, LTC site volunteers, and LTC management centres; IS included community leaders, friends/relatives, TV, print media, internet, and patient and family support groups. Support groups were classified as IS due to their peer-based and experiential nature, despite occasional professional or institutional ties. LTC care workers were excluded to avoid potential reverse causality, as they may be engaged after LTC use begins. Including them as FS could obscure the temporal relationship between information exposure and service uptake. Respondents could select multiple sources; FS and IS counts were calculated separately, with a maximum of 4 FS and 6 IS.

#### Lack of information

In Question A12 of the questionnaire, respondents selected reasons for not using LTC 2.0 services from 13 predefined options (multiple responses allowed). For this study, we defined an information gap as selecting any of the following: unawareness of LTC, lack of understanding of service content, or unfamiliarity with the application process. These items were pre-specified in the questionnaire to capture perceived informational barriers.

#### Covariates

The questionnaire collected data on three types of determinants for LTC service use: predisposing, need, and enabling factors. Predisposing factors included employer and care recipient such as gender, age, education, and living arrangement (with spouse, with others, or alone). Respondents also reported caregiving role (whether they were the primary caregiver) and duration of employing a migrant caregiver (≥ 3 years vs. <3 years). Care needs were assessed using eight tasks, including assistance with basic daily activities, using telephone and taking medications, housework, outdoor walking or activities, medical appointments, companionship, dementia and mental disability care, and special nursing care for behavioural problems and airway clearance. Each task item scored 1 (0–8 total), with care recipients grouped into four levels: ≤1, 2, 3, and ≥ 4 items. Care assistance needs were assessed using the same eight tasks as care needs, based on whether the migrant caregiver could handle each independently. One point was given for each task requiring additional help, with total scores grouped as in care needs; higher values indicated greater need for supplementary assistance.

Enabling factors included monthly household income, initially reported in New Taiwan Dollars (NTD), in ranges such as “No income,” “≤20,000 NTD,” “20,001–40,000,” and “≥200,001.” For analysis, income was grouped using a 60,000 NTD threshold. Household income was missing for 29 respondents (6.6%). To preserve sample size, we included these cases as a separate category in analyses, along with the groups of ≥ 60,000 NTD and < 60,000 NTD. Respondents also reported their financial status, classified as low-income or middle-low-income under the Public Assistance Act. Region (North, Central, South, East) was included as a contextual enabling variable affecting LTC access.

### Statistical analysis

Statistical analysis included descriptive statistics, with categorical variables shown as counts and percentages, and continuous variables as means and standard deviations. Chi-square tests examined associations between information sources and LTC use.

To test Hypothesis 1, univariate logistic regressions examined the separate effects of FS and IS on LTC utilisation. Multivariate models included both FS and IS concurrently as independent variables, allowing us to distinguish their associations while controlling for confounders. A sensitivity analysis reclassified LTC care workers as FS to evaluate potential bias.

To test Hypothesis 2, we first conducted bivariate χ² tests to screen covariates, retaining those with *p* < 0.2. These variables were then entered into multivariate logistic models using stepwise selection to identify predictors of FS and IS access.

For Hypothesis 3, additional logistic regression among non-users tested associations between FS/IS and perceived information gaps. All models reported odds ratios, 95% confidence intervals, and *p* < 0.05, using Stata v.17.

## Results

This study analysed 441 participants, of whom 49% used LTC services. As summarised in Table 1, LTC users and non-users differed across sociodemographic, caregiving, and financial characteristics. Compared to non-users, employers in LTC-using households were generally younger and more likely to be primary caregivers. Their care recipients were also younger, more likely to live with a spouse, and had higher care needs. Additionally, users were less likely to have employed migrant caregivers for over three years and more likely to report a monthly household income below 60,000 NTD.

**Table 1:** Characteristics of study participants, by user/non-user of long-term care services

Variables	Total (*n* = 441)	Use Status of Long-Term Care Services		*P* Value ^a^
		User (*n* = 216)	Non-user (*n* = 225)	
*Demographics*				
Employer gender, female (%)	229 (51.9)	120 (55.6)	109 (48.4)	0.135
Employer age, ≥ 60 y, n (%)	192 (43.5)	77 (35.6)	115 (51.1)	0.001
Employer education, tertiary or above, n (%)	262 (59.4)	134 (62.0)	128 (52.9)	0.271
Care recipient gender, female (%)	275 (62.4)	128(59.3)	147(65.3)	0.188
Care recipient age, n (%)				
< 70 y	58 (13.2)	33 (15.3)	25 (11.1)	0.005
70–79 y	67 (15.2)	41 (19.0)	26 (11.6)	
80–84 y	88 (20.0)	50 (23.1)	38 (16.9)	
≥ 85 y	228 (51.7)	92 (42.6)	136 (60.4)	
Care recipient education, ≥junior high school, n (%)	174 (39.5)	91 (42.1)	83 (36.9)	0.260
Living arrangement, n (%)				0.007
Living with spouse	127 (28.8)	77 (35.6)	50 (22.2)	
Living with other family members	213 (48.3)	96 (44.4)	117 (52.0)	
Living alone	101 (22.9)	43 (19.9)	58 (25.8)	
*Caregiving Characteristics*				
Employer as primary caregiver, n (%)	264 (59.9)	145 (67.1)	119 (52.9)	0.002
Care needs score (0–8), n (%)				< 0.001
0–1	44 (10.0)	5 (2.3)	39 (17.3)	
2	124 (28.1)	45 (20.8)	79 (35.1)	
3	224 (50.8)	133 (61.6)	91 (40.4)	
4+	49 (11.1)	33 (15.3)	16 (7.1)	
Care assistance score (0–8), n (%)				0.058
0–1	196 (44.4)	82 (38.0)	114 (50.7)	
2	104 (23.6)	55 (25.5)	49 (21.8)	
3	67 (15.2)	38 (17.6)	29 (12.9)	
4+	74 (16.8)	41 (19.0)	33 (14.7)	
Employing migrant caregiver ≥ 3 years, n (%)	326 (73.9)	140 (64.8)	186 (82.7)	< 0.001
*Financial Aspect*				
Monthly household income, n (%)				0.041
< 60,000 NTD	220 (49.9)	121 (56.0)	99 (44.0)	
≥ 60,000 NTD	192 (43.5)	83 (38.4)	109 (48.4)	
Missing	29 (6.6)	12 (5.6)	17 (7.6)	
Lower-middle or low-income household, n (%)	17 (3.9)	11 (5.1)	6 (2.7)	0.186
*Region*,* n (%)*				*0.883*
North	224 (50.8)	109 (50.5)	115 (51.1)	
Central	92 (20.9)	48 (22.2)	44 (19.6)	
South	97 (22.0)	45 (20.8)	52 (23.1)	
East	28 (6.3)	14 (6.5)	14 (6.2)	
*Information Sources*				
No. of formal sources (mean ± SD)	0.84 (± 0.80)	0.99 (± 0.74)	0.70 (± 0.83)	< 0.001
Accessed ≥ 1 formal source, n (%)	282 (63.9)	168 (77.8)	114 (50.7)	< 0.001
No. of informal sources (mean ± SD)	1.20 (± 1.15)	0.97 (± 1.08)	1.43 (± 1.17)	< 0.001
Accessed ≥ 1 informal source, n (%)	316 (71.7)	130 (60.2)	186 (82.7)	< 0.001

Data are expressed as number (%) or mean ± SD

^a^*P* values are based on Student t test for continuous variables and the χ^2^ test for categorical variables

Differences were also observed in LTC-related information behaviours. Users accessed FS more frequently and in greater numbers, while non-users more commonly relied on IS. Over three-quarters of users accessed at least one FS, compared to about half of non-users. Conversely, IS were used by a larger proportion of non-users, who also reported a higher average number of IS accessed.

**Fig. 1:**
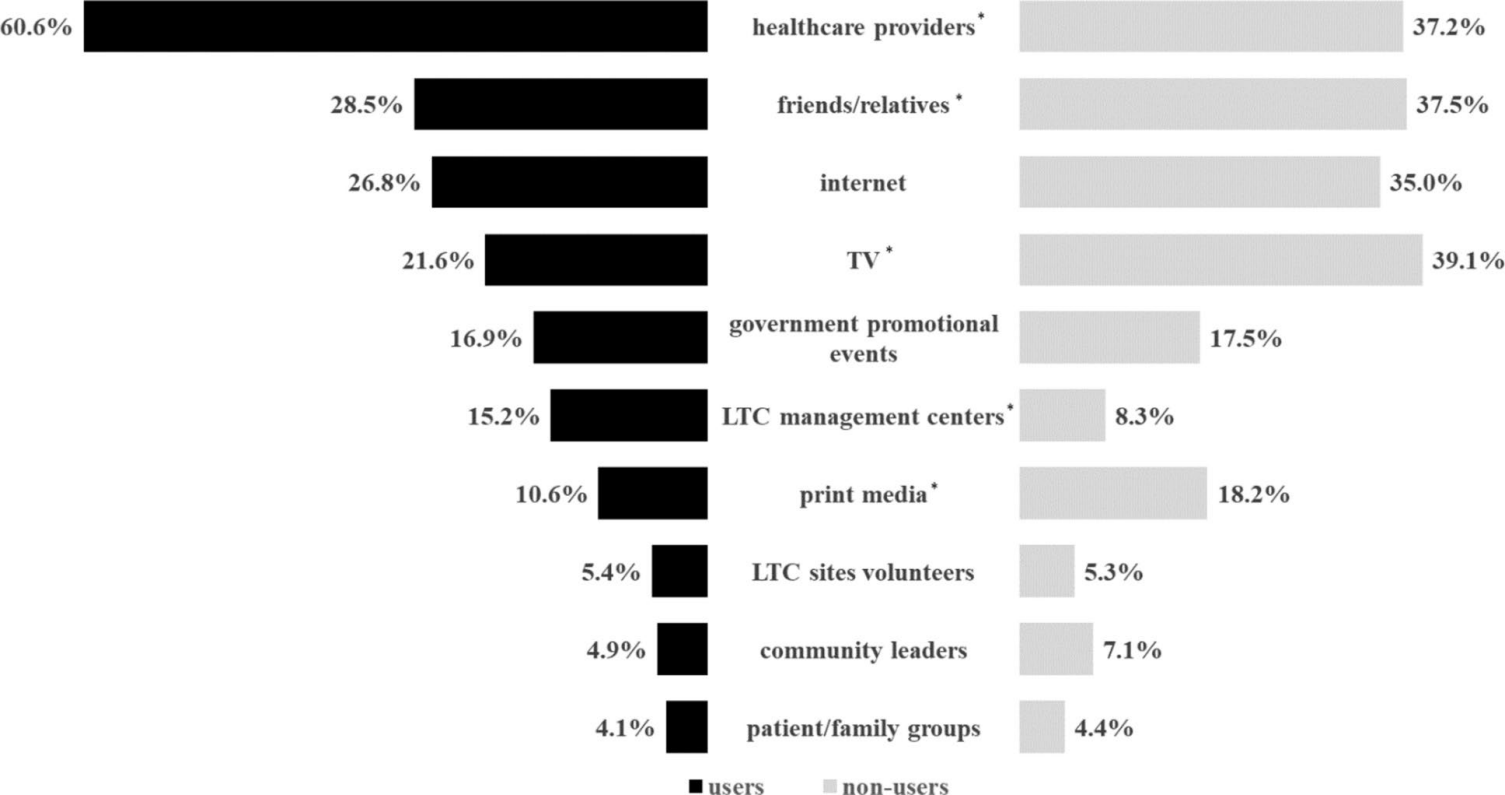
Comparison of long-term care information sources between LTC users and non-users. Note: This figure compares the percentages of LTC users and non-users by their information sources across 10 categories. Left black bars show users, right gray bars show non-users. Differences were assessed with χ² tests. *p < 0.05

The sources of LTC information differed between users and non-users. Figure 1 illustrates the specific sources used by each group: healthcare providers were the most frequently cited FS among users, whereas non-users more often relied on mass media and personal contacts, particularly television and print media.

Results of multivariate logistic regression (Table 2) indicate that each additional FS was associated with increased odds of LTC use, while each additional IS was associated with decreased odds. Other factors positively associated with LTC use included being a primary caregiver and having higher care needs. In contrast, older employer age, longer duration of employing migrant caregivers, and higher household income were linked to lower odds of LTC use. The sensitivity analysis showed nearly identical results, suggesting that excluding LTC care workers from FS might not introduce bias (see Additional file 3: SA Tables S1-S2).

**Table 2:** Regression analysis results for the use of long-term care services (*N* = 441)

Independent Variabless	Crude Model	Adjusted Model
	OR (95% CI)	OR (95% CI)
*Information Sources*		
No. of formal sources	1.62 (1.26–2.08)^***^	1.47 (1.10–1.96)^**^
No. of informal sources	0.68 (0.57–0.82)^***^	0.67 (0.54–0.82)^***^
*Demographics*		
Employer gender (ref.: male)		
Female	1.30 (0.90–1.90)	1.51 (0.95–2.39)
Employer age (ref.: < 60 y)		
≥ 60 y	0.55 (0.38–0.81)^**^	0.51 (0.31–0.82)^**^
Employer education (ref.: < tertiary)		
≥ tertiary	1.16 (0.79–1.69)	1.23 (0.75–2.02)
Care recipient gender (ref.: male)		
Female	0.78 (0.53–1.15)	0.96 (0.58–1.59)
Care recipient age (ref.: < 80 y)		
≥ 80 y	0.58 (0.38–0.88)^*^	0.95 (0.55–1.61)
Care recipient education (ref.: < junior high school)		
≥ junior high school	1.19 (0.81–1.74)	1.03 (0.61–1.74)
Living Arrangement (ref.: living with spouse)		
Living with other family members	0.54 (0.34–0.84)^**^	0.56 (0.32–0.99)^*^
Living alone	0.50 (0.29–0.85)^*^	0.63 (0.33–1.21)
*Caregiving Characteristics*		
Employer as primary caregiver (ref: no)		
Yes	1.78 (1.21–2.62)^***^	1.84 (1.15–2.95)^*^
Care need score (ref.: 0–1)		
2	5.4 (1.9–15.5)^**^	4.1 (1.3–12.6)^*^
3	13.2 (4.8–35.6)^***^	9.7 (3.2–29.2)^***^
4	16.9 (5.4–53.4)^***^	12.1 (3.5–42.7)^***^
Care assistance score (ref.: 0–1)		
2	1.66 (1.03–2.68)^*^	1.54 (0.87–2.74)
3	1.92 (1.10–3.34)^*^	1.81 (0.93–3.53)
4	1.72 (1.00-2.96)^*^	1.06 (0.55–2.04)
Employing migrant caregiver (ref.: < 3 y)		
≥ 3 y	0.37 (0.24–0.58)^***^	0.40 (0.24–0.68)^**^
*Financial Aspect*		
Monthly household income (ref.: < 60,000 NTD)		
≥ 60,000 NTD	0.60 (0.41–0.89)^*^	0.60 (0.37–0.99)^*^
Missing	0.57 (0.26–1.24)	0.46 (0.18, 1.14)
Lower-middle/low-income households (ref.: no)		
Yes	1.94 (0.71–5.26)	3.38 (0.87–13.19)
*Region* (ref.: North)		
Central	1.42 (0.90–2.25)	1.38 (0.81–2.35)
South	1.42 (0.89–2.28)	1.42 (0.80–2.53)
East	1.70 (0.59–4.90)	1.79 (0.52–6.15)

Values represent unadjusted or adjusted odds ratios (OR), with 95% confidence intervals (95% CI) in parentheses

Ref. denotes the reference group

^*^p < 0.05. ^**^p < 0.01. ^***^p < 0.001

Predictors of information source preferences are presented in Table 3. Access to FS was more likely among female employers, care recipients living with a spouse, and families who had employed a migrant caregiver for less than three years. In contrast, access to IS was more common among male employers, those with lower education levels, and families that had employed migrant caregivers for longer durations. Higher care needs were associated with greater use of FS and reduced use of IS.

**Table 3:** Regression analysis results on the information sources of long-term care services (*N* = 441)

Independent Variables	Information Sources	
	Formal Sources	Informal Sources
	OR (95% CI)	OR (95% CI)
*Demographics*		
Employer gender (ref.: male)		
Female	1.60 (1.06–2.42)^*^	0.45 (0.26–0.78)^**^
Employer education (ref.: < tertiary)		
≥ tertiary		0.54 (0.30–0.97)^*^
Care recipient gender (ref.: male)		
Female	1.38 (0.89–2.15)	
Living arrangement (ref.: living with spouse)		
living with others	0.46 (0.27–0.80)^**^	
living alone	0.34 (0.19–0.63)^***^	
*Caregiving Characteristics*		
Care need score (ref.: 0–1)		
2	3.20 (1.70–6.01)^***^	0.11 (0.02–0.53)^**^
3	3.45 (1.87–6.37)^***^	0.11 (0.02–0.50)^**^
4	5.62 (2.28–13.89)^***^	0.04 (0.01–0.20)^***^
Caregiving assistance score (ref.: 0–1)		
2		0.79 (0.39–1.61)
3		1.43 (0.54–3.76)
4		0.42 (0.21–0.89)^*^
Employing migrant caregiver (ref.: < 3 y)		
≥ 3 y	0.44 (0.26–0.76)^**^	3.43 (1.81–6.52)^***^
*Financial Aspect*		
Monthly household income (ref.: < 60,000 NTD)		
≥ 60,000 NTD		1.43 (0.79–2.57)
Missing		0.56 (0.21–1.44)

Multivariable logistic models were conducted using stepwise methods. Before engaging in stepwise regression, contingency analysis is utilized to initially select variables, retaining only those with a significance level of *p* < 0.20

Values represent adjusted odds ratios (OR), with 95% confidence intervals (95% CI) in parentheses

Ref. denotes the reference group

^*^p < 0.05. ^**^p < 0.01. ^***^p < 0.001

**Fig. 2:**
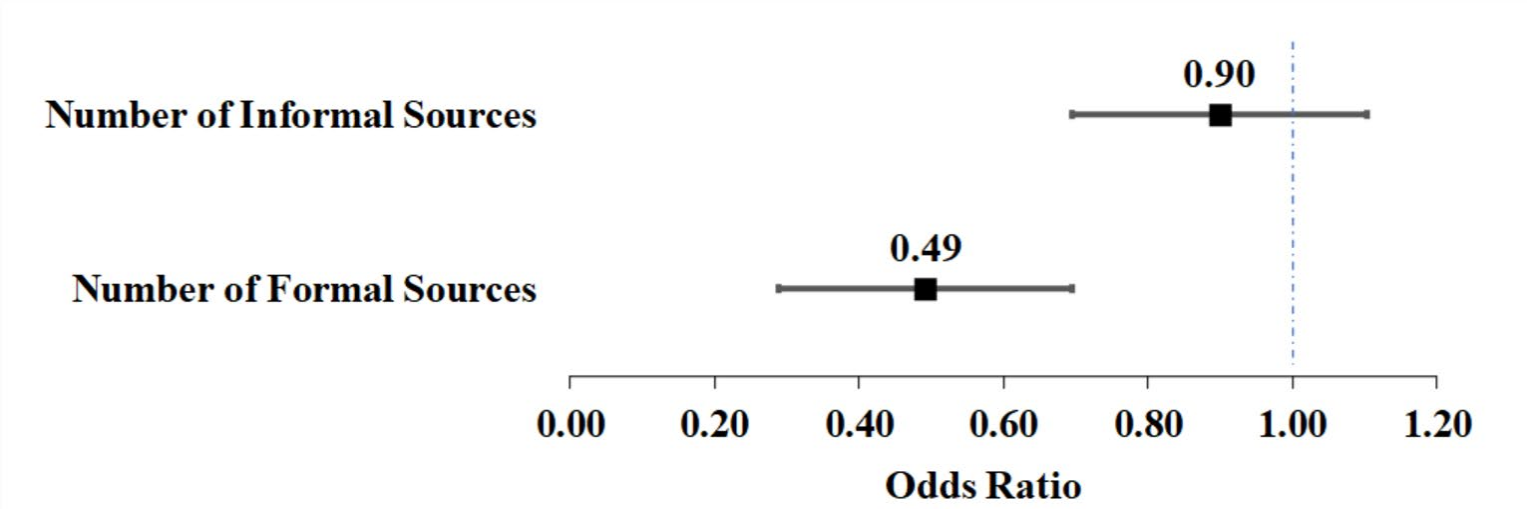
Impact of formal and informal information son the perceived information gap among non-users of long-term care. Note: This analysis uses multivariate logistic regression to explore the impact of formal and informal information source counts on non-users’ perceived information gap (*N* = 225). Adjustments include a range of characteristics of employers and care recipients. Odds ratios for each source count are represented by solid squares, and 95% confidence intervals by the extending lines

Among non-users, Fig. 2 shows that the likelihood of perceiving an information gap decreased significantly with the number of FS accessed, while no significant association was found for IS.

## Discussions

### Main findings

The study confirmed Hypothesis 1 by demonstrating that information sources significantly impact LTC utilisation, although the effects of formal and informal sources diverge. Specifically, FS are positively associated with increased LTC use, while IS correspond with a reduction in LTC utilisation. Consistent with Hypothesis 2, information sources were found to correlate with characteristics of employers and care recipients. We observed that access to FS was greater among female employers and care recipients with higher care needs, as well as those living with a spouse. Conversely, access to IS was higher among male employers and those with lower education levels, as well as care recipients with lower care needs. In addition, the longer the duration of employing a foreign caregiver, the less likely it is to obtain information from FS, and the more likely from IS. Hypothesis 3 was partially supported, showing that information sources impact perceived information gaps; FS were associated with a reduced likelihood of such gaps, whereas IS did not significantly affect them.

Our study aligns with Bradley et al.’s LTC utilisation framework, which emphasises the role of information, particularly source credibility and accessibility, in influencing LTC service use [35]. We investigated both formal and informal information sources, which was a novel aspect not explored in previous research. Our findings demonstrated that healthcare providers are the primary FS for LTC information, reflecting their pivotal role in health services [53]. Physicians significantly influenced patients LTC service utilization, ranking second only to family members [36]. However, studies show that physicians often lack familiarity with home- and community-based services (HCBS), including the types of services offered by LTC agencies, the eligibility criteria, and the referral processes, which may limit their ability to recommend appropriate options and lead them to default to institutional referrals [44, 54].

Despite these limitations, FS remain essential for informed decision-making. Their structured, credible, and policy-aligned nature supports confidence in service use [26, 46]. For example, healthcare providers, local LTC centres and government LTC hotlines may help clarify service eligibility, application procedures, and available options, thereby reducing informational and psychological barriers to access [44]. Our findings also showed that more FS were linked to fewer perceived information gaps. As often reliable and accurate sources, FS can reduce uncertainty and improve awareness of available LTC services [26].

Our study revealed diverse IS of LTC information, each accessed by approximately 20–30% of respondents, including family and friends, the internet, traditional media, and support groups. These sources often convey psychosocial or experiential content, such as caregiving stories, coping strategies, or personal impressions of services [49, 55]. The usefulness of IS depends on factors like perceived credibility, ease of access, and health literacy [56–58], but concerns about information accuracy are common, especially with online content [42], which offers immediacy and privacy when seeking information on sensitive issues [59].

Several mechanisms may explain the negative association between IS and LTC use observed. First, IS are often incomplete or inaccurate; social media, for instance, contains widespread misinformation that blurs the line between fact and fiction [42]. Second, they may delay engagement with services by shaping perceptions based on anecdotal experiences rather than professional guidance [28]. Third, unlike FS that provide tailored guidance, IS lack comprehensiveness and personalization, making it more difficult for individuals to act on such information effectively, which in turn may hinder LTC use [26]. Finally, IS are often passively received, such as through TV or print media, and may lack sufficient clarity. Individuals may struggle to interpret or apply such information, and may lack the motivation to initiate service use [43].

For non-users in particular, IS were not associated with perceived information gaps. This may be because many of these individuals did not perceive a need for LTC, especially if care needs were already met by migrant caregivers, who often serve as substitutes for formal care [18]. Without a felt need, they are less likely to actively seek or evaluate LTC information from IS, even when exposed to it.

As care needs escalate, families increasingly rely on FS over IS since FS provide professional guidance that is unavailable from family or friends. Source choice hinges on perceived service benefits. Families with higher care needs place greater value on professional care and consequently have a higher demand for its information. A study in Singapore found home nursing services to be the most favoured LTC service because they meet the needs for care provided by professionally trained personnel and in-home care [60]. Similarly, in Taiwan, the LTC 2.0 system offers professional guidance for those with additional care needs, such as nursing care, nutritional care, and rehabilitation. By contrast, families may not seek formal LTC services and relevant information when additional care needs are minimal.

This study investigated how care recipients’ and family characteristics influence information-seeking and source preferences. Studies indicate that women and younger individuals more actively seek health information [40, 41]. Higher educational attainment correlates with obtaining health information from magazines, the internet, and books, rather than television [51]. Sociodemographic factors like age, gender, education, employment status, and immigration status are linked to professional, government, and online sources, but not personal connections [39]. This study contributes to existing literature by examining FS versus IS. The results demonstrated that women are more likely to use FS and less likely to use IS. Those with higher education levels tend to rely on IS lesser. Additionally, care recipients not living with a spouse or living alone are less inclined to use FS, possibly owing to limited family support and information access [52].

Families that employ migrant caregivers for longer durations are less likely to access FS and use LTC 2.0 services. In one study on Taiwanese families employing migrant caregivers, family members, primarily women, took charge of hiring and coordinating with live-in caregivers, often making them part of the household routine [18]. Over time, this allowed families to manage care needs in a more flexible, individualised manner, potentially reducing reliance on formal LTC services. Meanwhile, these families were also found to have stronger family networks [14], suggesting the presence of more available informal caregivers, which may further reduce the perceived need for formal LTC services. These factors together may lower engagement with both FS and LTC 2.0 services.

Contrary to prior studies suggesting that financial constraints influence the use of FS [38], our study found no significant association between household income and the use of either FS or IS. This finding may partially reflect Taiwan’s universal National Health Insurance system, which has improved access to healthcare providers, particularly for low- and middle-income older adults [61]. Under the LTC 2.0 policy, co-payment waivers for low-income households further reduce financial barriers to service use and information access [10]. IS use also showed no income-based differences, possibly due to their low cost, ease of access, and passive dissemination through daily interactions or media, which may make them less sensitive to financial constraints. These mechanisms may help explain the absence of income-based disparities in accessing LTC information.

Our study excluded residential region from the final FS and IS models (Table 3) because it did not meet the stepwise selection threshold (*p* < 0.2). This may reflect the use of broad regional categories, which could obscure local disparities in information access. In contrast, studies in the United States have reported significant rural–urban differences in access to health information, with rural residents having significantly lower access to formal sources such as specialist doctors [50]. Future studies should adopt finer geographic measures and assess actual proximity to LTC information.

### Policy implications

Our study highlights how the information sources accessed by individuals and families significantly influence their decisions regarding LTC service usage. FS have proven effective in improving LTC utilisation. Therefore, it is essential for policymakers further to enhance the influence and reach of these sources by strategies such as expanding their accessibility through information and communication technologies (ICT), for example, mobile applications and online service portals, which facilitate connection to community resources and support services, helping caregivers navigate available services more effectively [62]. Another strategy is investing in LTC navigator programmes, which can assist care recipients and families in navigating care transitions and accessing appropriate services. These navigators help remove asymmetry of information that prevents clients from engaging in their care and understanding the options available to them [63]. In addition, strengthening education and training for physicians and other health professionals on LTC referral pathways and the LTC 2.0 system could improve the integration of LTC information into clinical practice, including discharge planning [44, 64].

Furthermore, community LTC sites are crucial for bridging the information gap between community residents and LTC services. For instance, within the LTC 2.0 system, Tier-C stations (local LTC facilities) offer preventive disability-delaying programmes, temporary respite care and meal services for community-dwelling older adults who are frail or disabled [9]. These stations are the most minor operational units and could be housed in community-based settings such as coffee shops, cafeterias, drug stores, gyms, or community centres—often termed ‘the third place ’ [65]. These locations are frequently visited by older adults and caregivers, making them accessible venues for LTC-related information. For example, the ElderSmile programme in New York City utilised community-based third places, such as senior centres, to deliver oral health education and chronic disease screenings [66]. The programme demonstrated that socially familiar and accessible venues can effectively engage older adults and facilitate the dissemination of health information, suggesting the potential of third places as trusted settings for promoting LTC awareness and improving access to relevant services and information​.

On the other hand, this study revealed that IS negatively impact LTC service usage without a direct link to perceived information gaps. This suggests that while IS often provide information, its accuracy may be questionable. When trusted FS fail to engage with the public, misinformation may spread through IS [42]. Strengthening FS, particularly in community or third-place settings, can improve direct access to accurate information and enhance the reliability of IS by influencing the content disseminated through these channels. Therefore, LTC authorities should work with community leaders and key stakeholders to ensure that outreach campaigns convey accurate information and effectively promote public awareness of LTC [67]. Further research is needed to explore which types of strategies, when conveyed through IS, may reduce families’ reluctance to use LTC services.

Beyond Taiwan, these findings have implications for countries that rely heavily on migrant live-in caregivers amid rapidly aging populations and care workforce shortages [68]. Enhancing formal LTC information sources may encourage families to use services such as respite care, thereby improving the psychological well-being of caregivers who bear intensive care burdens. Information campaigns that raise awareness of LTC options and promote flexible HCBS may further reduce reliance on migrant live-in caregivers and support more sustainable care arrangements [68].

### Limitations

This study has some limitations. First, because the study focused on families employing migrant caregivers, the findings are internally valid for this group but may not be generalizable. Extrapolating these results to broader populations could lead to biased estimates of associations between information sources and LTC use. Specifically, the positive relationship observed between FS and LTC use may be underestimated, as families already relying on live-in caregivers might have lower incentive to seek formal LTC 2.0 services despite accessing FS. Conversely, the negative association between IS and LTC use may have been overestimated. Families without migrant caregivers might face greater care burdens and be more inclined to seek accurate information from FS, even when IS are available. Therefore, the observed deterrent effect of IS in our findings may reflect characteristics specific to families employing migrant caregivers.

Second, the survey respondents, primarily the employers, may not be the primary caregivers, potentially leading to incomplete knowledge about LTC service usage and introducing recall biases. Third, the cross-sectional design limits causal inference. Individuals inclined to use LTC services may be more likely to seek FS, rather than FS directly promoting LTC use. Likewise, reliance on IS may reflect distrust in FS, or further discourage its use by reinforcing existing perceptions. These dynamics among LTC intention, FS, and IS cannot be clarified in this study, underscoring the need for alternative designs to explore causality.

## Conclusions

Comparing LTC users and non-users among families employing migrant care workers, this study identified a positive association between LTC service usage and access to FS of information, as well as a negative association with IS. These information sources correlate with the characteristics of care recipients and their families. We recommend that LTC authorities improve FS accessibility for both families and the public, while implementing strategies to mitigate the negative impact of IS on LTC services.

## Supplementary Information

Supplementary Material 1.

Supplementary Material 2.

Supplementary Material 3.

## Data Availability

The datasets generated and analysed during the current study are not publicly available due to licensing agreements but are available from the corresponding author on reasonable request.

## Abbreviations

LTC: Long-term care
FS: Formal sources
IS: Informal sources
NTD: New Taiwan Dollars
HCBS: Home- and community-based services
LTCC: Long-term Care Centre
MOL: Ministry of Labour
MOHW: Ministry of Health and Welfare
