# Supplementary material for: Information sources as determinants of use of formal long-term care: a cross-sectional study in Taiwan
Source: BMC Health Serv Res. 2025 Jul 3;25:910. doi: 10.1186/s12913-025-12814-6 (PMC12225371; doi:10.1186/s12913-025-12814-6)
Supplement: Supplementary file 1 — Supplementary Material 1. [file 12913_2025_12814_MOESM1_ESM.docx]

Questionnaire Survey on the Use of Long-Term Care Services by Households Employing Migrant Care Workers

Dear interviewee:

The National Health Research Institutes was commissioned by the Department of Long-Term Care, Ministry of Health and Welfare to undertake the “Research and Analysis Project on Bridging Households Employing Migrant Live-in Care Workers with the Long-Term Care 2.0 (LTC 2.0) Service System”. This project seeks to understand the status of LTC 2.0 use by households employing migrant care workers, the quality of care provided by the migrant care workers employed, and the views of these households on long-term care policies. All interviewees are kindly requested to respond based on your actual situation.

This survey will be completed with the assistance of telephone interviewers. The researchers will ensure the confidentiality of any personally identifiable information, and no personal information will be presented in the research results. Thank you for your help!

If you have any questions about this project, please contact ⬛⬛⬛⬛ at the Institute of Population Health Sciences, National Health Research Institute (telephone no.: 037-206166 ext. ⬛⬛⬛).

I wish you success, health and happiness for the future.

Yours sincerely, Chiou Hung-Yi, Director of the Institute of Population Health Sciences, National Health Research Institutes

01. Does your household currently employ a migrant live-in care worker?

(1) □Yes (2) □No **(end interview)**

02. Are you the employer?

(1) □Yes (2) □No

03. Apart from the migrant care worker, are you the primary or secondary caregiver in the household, or not involved in caregiving?

(1) □Primary caregiver **(skip to Q04)** (2) □Secondary caregiver **(skip to Q04)** (3) □Not involved in caregiving **(continue to Q03.1)**

(03.1) Do you understanding the care situation of the care recipient?

(03.1-1) □Yes

(03.1-2) □No **(end interview, or ask the interviewee to recommend someone who understands the care situation)**

04. What is your gender?

(1) □Male (2) □Female

05. What is your age?

(1) □20–39 years (2) □40–49 years (3) □50–59 years (4) □60–69 years (5) □70 years and above

06. What is your education level?

(1) □Illiterate (2) □Home-schooled (3) □Graduated/attended elementary school (4) □Graduated/attended junior high school (5) □Graduated/attended senior high school (6) □Junior college/university and above

07. How do you refer to the care recipient?

(1) □(Grand)Father/Mother (including in-laws) (2) □Spouse (including cohabitants)

(3) □(Grand)Son/Daughter (including in-laws) (4) □Sibling (including in-laws)

(5) □Other relatives and friends (6) □Other

08. How long have you employed migrant care workers in your household? (Please state length of time starting from the first worker, and approximate start time)

A total of 　　years 　　months. (Starting from 　　 year 　　month)

09. Following from the previous question, how long has the current migrant care worker cared for the care recipient? Current migrant live-in care worker has cared for the care recipient for 　　years 　　months.

Part I　LTC 2.0 Use by Households Employing Migrant Care Workers

LTC 2.0: With the aim of providing a diverse range of care services and establishing a community-based service system to cope with the problems arising from an aging society, the Executive Yuan approved the LTC 2.0 in 2016. Its service content includes personal and professional care, transportation services, assistive devices, barrier-free home modification, respite care services, etc.

**The interviewer should confirm if the interviewee list is □A (currently using LTC 2.0) or □B (not using LTC 2.0).**

1. **Prior to employing migrant care workers**, did the care recipient or family members use any LTC 2.0 services? (**Select all that apply.** The interviewer can provide prompts in the following order)

(1) □Home services (A3) (2) □Day-care services (A4)

(3) □Professional care (A5) (4) □Transportation services (A6)

(5) □Auxiliary appliance services (A7) (6) □ Barrier-free home environment modification (A8)

(7) □Respite care services (A9) (8) □Long-term care stations in alleys and lanes (Tier C stations) (A10)

(9) □Home-visit bathing car (A11) (10) □Did not use LTC 2.0 services

(11) □Other:

1. **After employing migrant care workers**, did the care recipient or family members use any of the following LTC 2.0 services? (**Select all that apply.** The interviewer can provide prompts in the following order)

(1) □Professional care (A5) (2) □Transportation services (A6)

(3) □Auxiliary appliance services (A7) (4) □Barrier-free home environment modification (A8)

(5) □Respite care services (A9) (6) □Long-term care stations in alleys and lanes (Tier C stations) (A10)

(7) □Home-visit bathing car (A11) (8) □Other:

(9) □Did not use LTC 2.0 services

A2.1 What LTC 2.0 services do the care recipient or family members **currently (within the past three months)** use? (The interviewer should confirm whether the responses to QA2.1 also appear in QA2)

(1) □Professional care (A5) (2) □Transportation services (A6)

(3) □Auxiliary appliance services (A7) (4) □Barrier-free home environment modification (A8)

(5) □Respite care services (A9) (6) □Long-term care stations in alleys and lanes (Tier C stations) (A10)

(7) □Home-visit bathing car (A11) (8) □Other:

(9) □Do not use LTC 2.0 services (after confirming the interviewee is on List **B**, skip to QA12)

**If any interviewee on List A selects “(9) Do not use LTC 2.0 services” for QA2.1, please end the interview;**

**if any interviewee on List B selects any option from (1) to (8) for QA2.1, please end the interview.**

1. This item should be answered based on whether “**(1) Home services”** was selected in QA1:

(1) □Yes **(continue to QA3.1)** (2) □No **(skip to QA4)**

(A3.1) Are you satisfied with **“home services”**?

(3.1-1) □Very satisfied **(skip to QA4)**

(3.1-2) □Satisfied **(skip to QA4)**

(3.1-3) □Neutral **(skip to QA4)**

(3.1-4) □Dissatisfied **(continue to QA3.2)**

(3.1-5) □Very dissatisfied **(continue to QA3.2)**

(A3.2) Why are you dissatisfied with **“home services”**? **(Select all that apply)**

(3.2-1) □Confusing/troublesome application process

(3.2-2) □Copayment too high to afford

(3.2-3) □Disliked by care recipient

(3.2-4) □Inadequate benefit cap

(3.2-5) □Poor service quality

(3.2-6) □Unsuitable timing

(3.2-7) □Service content does not meet expectations

(3.2-8) □Too few (or unavailable) care stations nearby

(3.2-9) □Unable to freely choose care attendants (home care attendants)

(3.2-10) □Other

1. This item should be answered based on whether **“(2) Day-care services”** was selected in QA1:

(1) □Yes **(continue to QA4.1)** (2) □No **(skip to QA5)**

(A4.1) Are you satisfied with **“day-care services”?**

(4.1-1) □Very satisfied **(skip to QA5)**

(4.1-2) □Satisfied **(skip to QA5)**

(4.1-3) □Neutral **(skip to QA5)**

(4.1-4) □Dissatisfied **(continue to QA4.2)**

(4.1-5) □Very dissatisfied **(continue to QA4.2)**

(A4.2) Why are you dissatisfied with **“day-care services” (Select all that apply)**

(4.2-1) □Confusing/troublesome application process

(4.2-2) □Copayment too high to afford

(4.2-3) □Disliked by care recipient

(4.2-4) □Inadequate benefit cap

(4.2-5) □Poor service quality

(4.2-6) □Unsuitable timing

(4.2-7) □Service content does not meet expectations

(4.2-8) □Too few (or unavailable) care stations nearby

(4.2-9) □Other

1. This item should be answered based on whether **“(1) Professional care”** was selected in QA2.1:

(1) □Yes **(skip to QA5.2)** (2) □No **(continue to QA5.1)**

(A5.1) What are your reasons for not using **“professional care”**? **(Select all that apply, then skip to QA6)**

(5.1-1) □Unaware of this service

(5.1-2) □Unsure about service content

(5.1-3) □Do not understand service application process

(5.1-4) □Copayment too high to afford

(5.1-5) □Disliked by care recipient

(5.1-6) □Inadequate benefit cap

(5.1-7) □Poor service quality (effectiveness)

(5.1-8) □Unsuitable timing

(5.1-9) □Not eligible for this service

(5.1-10) □Unable to use due to restrictions in regulation (e.g. case will be closed without progress)

(5.1-11) □Other

(A5.2) Are you satisfied with **“professional care”**?

(5.2-1) □Very satisfied **(skip to QA6)**

(5.2-2) □Satisfied **(skip to QA6)**

(5.2-3) □Neutral **(skip to QA6)**

(5.2-4) □Dissatisfied **(continue to QA5.3)**

(5.2-5) □Very dissatisfied **(continue to QA5.3)**

(A5.3) Why are you dissatisfied with **“professional care”**? **(Select all that apply)**

(5.3-1) □Confusing/troublesome application process

(5.3-2) □Copayment too high to afford

(5.3-3) □Disliked by care recipient

(5.3-4) □Inadequate benefit cap

(5.3-5) □Poor service quality (effectiveness)

(5.3-6) □Unsuitable timing

(5.3-7) □Service content does not meet expectations

(5.3-8) □Unable to freely choose care attendants

(5.3-9) □Other

1. This item should be answered based on whether “**(2) Transportation services”** was selected in QA2.1:

(1) □Yes **(skip to QA6.2)** (2) □No **(continue to QA6.1)**

(A6.1) What are your reasons for not using **“transportation services”**? **(Select all that apply, then skip to QA7)**

(6.1-1) □Unaware of this service

(6.1-2) □Unsure about service content

(6.1-3) □Do not understand service application process

(6.1-4) □Copayment too high to afford

(6.1-5) □Disliked by care recipient

(6.1-6) □Inadequate benefit cap

(6.1-7) □Poor service quality (e.g.: driver with bad attitude, vehicle in poor condition, etc.)

(6.1-8) □Unsuitable timing

(6.1-9) □Not eligible for this service

(6.1-10) □Unable to book this service

(6.1-11) □Other

(A6.2) Are you satisfied with **“transportation services”**?

(6.2-1) □Very satisfied **(skip to QA7)**

(6.2-2) □Satisfied **(skip to QA7)**

(6.2-3) □Neutral **(skip to QA7)**

(6.2-4) □Dissatisfied **(continue to QA6.3)**

(6.2-5) □Very dissatisfied **(continue to QA6.3)**

(A6.3) Why are you dissatisfied with **“transportation services”**? **(Select all that apply)**

(6.3-1) □Confusing/troublesome application process

(6.3-2) □Copayment too high to afford

(6.3-3) □Disliked by care recipient

(6.3-4) □Inadequate benefit cap

(6.3-5) □Poor service quality (e.g.: driver with bad attitude, vehicle in poor condition, etc.)

(6.3-6) □Unsuitable timing

(6.3-7) □Service content does not meet expectations

(6.3-8) □Very difficult to book this service

(6.3-9) □Other

1. This item should be answered based on whether “**(3) Auxiliary appliance services”** was selected in QA2.1:

(1) □Yes **(skip to QA7.2)** (2) □No **(continue to QA7.1)**

(A7.1) What are your reasons for not using **“auxiliary appliance services”**? **(Select all that apply, then skip to QB8)**

(7.1-1) □Unaware of this service

(7.1-2) □Unsure about service content

(7.1-3) □Do not understand service application process

(7.1-4) □Copayment too high to afford

(7.1-5) □Disliked by care recipient

(7.1-6) □Inadequate benefit cap

(7.1-7) □Poor service quality

(7.1-8) □Hire period of assistive devices unable to meet requirements

(7.1-9）□Assistive devices unable to meet needs of care recipient

(7.1-10) □Other

(A7.2) Are you satisfied with **“auxiliary appliance services”**?

(7.2-1) □Very satisfied **(skip to QA8)**

(7.2-2) □Satisfied **(skip to QA8)**

(7.2-3) □Neutral **(skip to QA8)**

(7.1-4) □Dissatisfied **(continue to QA7.3)**

(7.2-5) □Very dissatisfied **(continue to QA7.3)**

(A7.3) Why are you dissatisfied with **“auxiliary appliance services”**? **(Select all that apply)**

(7.3-1) □Confusing/troublesome application process

(7.3-2) □Copayment too high to afford

(7.3-3) □Disliked by care recipient

(7.3-4) □Inadequate benefit cap

(7.3-5) □Poor service quality

(7.3-6) □Service content does not meet expectations

(7.3-7) □Hire period of assistive devices unable to meet requirements

(7.3-8）□Assistive devices unable to meet needs of care recipient

(7.3-9) □Other

1. This item should be answered based on whether “**(4) Barrier-free home environment modification”** was selected in QA2.1:

(1) □Yes **(skip to QA8.2)** (2) □No **(continue to QA8.1)**

(A8.1) What are your reasons for not using **“barrier-free home environment modification”**? **(Select all that apply, then skip to QB9)**

(8.1-1) □Unaware of this service

(8.1-2) □Unsure about service content

(8.1-3) □Do not understand service application process

(8.1-4) □Copayment too high to afford

(8.1-5) □Disliked by care recipient

(8.1-6) □Inadequate benefit cap

(8.1-7) □Poor service quality

(8.1-8) □Other

(A8.2) Are you satisfied with **“barrier-free home environment modification”**?

(8.2-1) □Very satisfied **(skip to QB9)**

(8.2-2) □Satisfied **(skip to QB9)**

(8.2-3) □Neutral **(skip to QB9)**

(8.2-4) □Dissatisfied **(continue to QB8.3)**

(8.2-5) □Very dissatisfied **(continue to QB8.3)**

(A8.3) Why are you dissatisfied with **“barrier-free home environment modification”**? (Select all that apply)

(8.3-1) □Confusing/troublesome application process

(8.3-2) □Copayment too high to afford

(8.3-3) □Disliked by care recipient

(8.3-4) □Inadequate benefit cap

(8.3-5) □Poor service quality

(8.3-6) □Service content does not meet expectations

(8.3-7) □Other

1. This item should be answered based on whether **“(5) Respite care services”** was selected in QA2.1:

(1) □Yes **(skip to QA9.2)** (2) □No **(continue to QA9.1)**

(A9.1) What are your reasons for not using **“respite care services”**? **(Select all that apply, then skip to QB10)**

(9.1-1) □Unaware of this service

(9.1-2) □Unsure about service content

(9.1-3) □Do not understand service application process

(9.1-4) □Copayment too high to afford

(9.1-5) □Disliked by care recipient

(9.1-6) □Inadequate benefit cap

(9.1-7) □Poor service quality

(9.1-8) □Insufficient flexibility of service availability (maximum of 10 hours a day)

(9.1-9) □Too few (or unavailable) care stations nearby

(9.1-10) □Unable to book care attendants

(9.1-11) □Unsuitable timing

(9.1-12) □Other

(A9.2) Are you satisfied with **“respite care services”**?

(9.2-1) □Very satisfied **(skip to QA10)**

(9.2-2) □Satisfied **(skip to QA10)**

(9.2-3) □Neutral **(skip to QA10)**

(9.2-4) □Dissatisfied **(continue to QA9.3)**

(9.2-5) □Very dissatisfied **(continue to QA9.3)**

(A9.3) Why are you dissatisfied with **“respite care services”**? **(Select all that apply)**

(9.3-1) □Confusing/troublesome application process

(9.3-2) □Copayment too high to afford

(9.3-3) □Disliked by care recipient

(9.3-4) □Inadequate benefit cap

(9.3-5) □Poor service quality

(9.3-6) □Insufficient flexibility of service availability (maximum of 10 hours a day)

(9.3-7) □Too few (or unavailable) care stations nearby

(9.3-8) □Service content does not meet expectations

(9.3-9) □Unable to freely choose care attendants (home care attendants)

(9.3-10) □Unsuitable timing

(9.3-11) □Other

1. This item should be answered based on whether “**(6) Long-term care stations in alleys and lanes (Tier C stations)”** was selected in QA2.1:

(1) □Yes **(skip to QA10.2)** (2) □No **(continue to QA10.1)**

(A10.1) What are your reasons for not using **“long-term care stations in alleys and lanes (Tier C stations)”**? **(Select all that apply, then skip to QB11)**

(10.1-1) □Unaware of this service

(10.1-2) □Unsure about service content

(10.1-3) □Do not understand service application process

(10.1-4) □Copayment too high to afford

(10.1-5) □Disliked by care recipient

(10.1-6) □Poor service quality

(10.1-7) □Unsuitable timing

(10.1-8) □Not eligible for this service

(10.1-9) □No Tier C stations nearby

(10.1-10) □Other

(A10.2) Are you satisfied with **“long-term care stations in alleys and lanes (Tier C stations)”**?

(10.2-1) □Very satisfied **(skip to QA11)**

(10.2-2) □Satisfied **(skip to QA11)**

(10.2-3) □Neutral **(skip to QA11)**

(10.2-4) □Dissatisfied **(continue to QA10.3)**

(10.2-5) □Very dissatisfied **(continue to QA10.3)**

(A10.3) Why are you dissatisfied with **“long-term care stations in alleys and lanes (Tier C stations)”**? **(Select all that apply)**

(10.3-1) □Confusing/troublesome application process

(10.3-2) □Copayment too high to afford

(10.3-3) □Disliked by care recipient

(10.3-4) □Poor service quality

(10.3-5) □Unsuitable timing

(10.3-6) □Service content does not meet expectations

(10.3-7) □No Tier C stations nearby

(10.3-9) □Other

1. This item should be answered based on whether **“(7) Home-visit bathing car”** was selected in QA2.1:

(1) □Yes **(skip to QA11.2)** (2) □No **(continue to QA11.1)**

(B11.1) What are your reasons for not using **“home-visit bathing car”**? **(Select all that apply, then skip to QB13)**

(11.1-1) □Unaware of this service

(11.1-2) □Unsure about service content

(11.1-3) □Do not understand service application process

(11.1-4) □Copayment too high to afford

(11.1-5) □Disliked by care recipient

(11.1-6) □Inadequate benefit cap

(11.1-7) □Poor service quality

(11.1-8) □Unsuitable timing

(11.1-9) □Not eligible for this service

(11.1-10) □Unable to freely choose care attendants

(11.1-11) □Other

(A11.2) Are you satisfied with **“home-visit bathing car”**?

(11.2-1) □Very satisfied **(skip to QA13)**

(11.2-2) □Satisfied **(skip to QA13)**

(11.2-3) □Neutral **(skip to QA13)**

(11.2-4) □Dissatisfied **(continue to QA11.3)**

(11.2-5) □Very dissatisfied **(continue to QA11.3)**

(A11.3) Why are you dissatisfied with **“home-visit bathing car”**? **(Select all that apply, then skip to QB13)**

(11.3-1) □Confusing/troublesome application process

(11.3-2) □Copayment too high to afford

(11.3-3) □Disliked by care recipient

(11.3-4) □Inadequate benefit cap

(11.3-5) □Poor service quality

(11.3-6) □Unsuitable timing

(11.3-7) □Service content does not meet expectations

(11.3-8) □Unable to freely choose care attendants

(11.3-9) □Other

1. What are your reasons for not using LTC 2.0 services? **[Interviewees who have responded to QB3–B11 need no answer this item]** **(Select all that apply)**

(1) □Unaware of LTC 2.0 services

(2) □Unsure about LTC 2.0 service content

(3) □Do not understand application process for LTC 2.0 services

(4) □Copayment too high to afford

(5) □Disliked by care recipient

(6) □Inadequate benefit cap

(7) □Poor service quality

(8) □Insufficient flexibility of service availability

(9) □Service content does not meet expectations

(10) □Not eligible for these services

(11) □Personnel unable to provide home care services in time

(12) □Unsuitable timing

(13) □Too few (or unavailable) care stations nearby

(14) □Other

1. Overall, which of the following LTC 2.0 services do you think are more helpful to the care recipient or their family members? **(Select all that apply)**

(1) □Home services (2) □Day-care services

(3) □Professional care (4) □Transportation services

(5) □Auxiliary appliance services (6) □ Barrier-free home environment modification

(7) □Respite care services (8) □Long-term care stations in alleys and lanes (Tier C stations)

(9) □Home-visit bathing car (10) □Other:

1. Where did you hear about long-term care services? **(Select all that apply)**

(1) □Government promotional activities (2) □Village/neighborhood leaders (3) □Family and friends (4) □Television (5) □Print media (6) □Internet

(7) □Volunteers at the LTC community sites (8) □Patient/family support groups

(9) □Care attendants (10) □Medical care institutions (including public health centers) (11) □Long-term care management center (12) □Other

1. How much copayment are you willing to pay in total each month for long-term care services? (Excluding costs of migrant care workers)

(1) □NT$ 3,000 and below (2) □NT$ 3,001–5,000 (3) □NT$ 5,001–10,000

(4) □NT$ 10,001–15,000 (5) □NT$ 15,001–20,000 (6) □NT$ 20,001–25,000

(7) □NT$ 25,000 and above

Part II　Quality of Migrant Live-in Care Workers

1. We will ask you a few questions in the next section. Please assess your level of satisfaction toward the performance of migrant care workers based on how you actually feel: **[**Please respond by stating if you are **“very satisfied, satisfied, neutral, dissatisfied, or very dissatisfied”]**

| Item | Very satisfied | Satisfied | Neutral | Dissatisfied | Very dissatisfied |
| --- | --- | --- | --- | --- | --- |
| B1.1 Overall performance | □ | □ | □ | □ | □ |
| B1-2. Care skills | □ | □ | □ | □ | □ |
| B1-3. Language ability | □ | □ | □ | □ | □ |
| B1-4. Work attitude | □ | □ | □ | □ | □ |
| B1-5. Work emotions | □ | □ | □ | □ | □ |
| B1-6. Work efficiency | □ | □ | □ | □ | □ |
| B1-7. Hygiene habits | □ | □ | □ | □ | □ |

1. Next, I will list several work items. Please respond based on your actual conditions about whether these items can be completed independently by migrant care workers, and assess their proficiency with these items. [Please respond by stating if they are “**Highly proficient, proficient, average, not proficient, not proficient at all”**]

| Code | Care work items | Note | Can be completed independently by migrant care worker  (without assistance from other family caregivers) | | | Caregiving skills and proficiency of migrant care workers | | | | |
| --- | --- | --- | --- | --- | --- | --- | --- | --- | --- | --- |
|  |  |  | Yes | No | Care recipient has no such need | Highly proficient | Proficient | Average | Not proficient | Not proficient at all |
| 1 | Basic care | Feeding, bathing, personal hygiene, dressing and undressing, toileting, mobility, stair-climbing | □ | □ | □ | □ | □ | □ | □ | □ |
|  |  | Using the telephone, taking medications | □ | □ | □ | □ | □ | □ | □ | □ |
| 2 | Housework | Grocery shopping, cooking, housekeeping, doing laundry, managing finances (bill payments) | □ | □ | □ | □ | □ | □ | □ | □ |
| 3 | Going outdoors for daily activities | Outdoor activities (taking walks), regular rehabilitation | □ | □ | □ | □ | □ | □ | □ | □ |
|  |  | Accompaniment to medical appointments (registering for medical consultation and collecting prescriptions) | □ | □ | □ | □ | □ | □ | □ | □ |
| 4 | Companionship care | Psychological support, chatting with care recipient, listening to their concerns | □ | □ | □ | □ | □ | □ | □ | □ |
| 5 | Advance care | Dementia care and mental disability care | □ | □ | □ | □ | □ | □ | □ | □ |
|  |  | Special care needs (behavioral problems, airway clearance etc.) | □ | □ | □ | □ | □ | □ | □ | □ |

1. If the migrant live-in care worker is away on leave, who will usually care for the care recipient **(Select all that apply)**

(1) □Family members (2) □Relatives or friends (3) □Local care worker (care attendant) (4) □Send care recipient to day-care center (5) □Apply for government respite care services

(6) □No issues with leave (7) □Other

Part III　Policy Suggestions

1. In what ways do you think future policies should improve the skills of migrant live-in care workers **(Open-ended response; the interviewer should encourage the interviewee to state at least one method)**
2. Do you support the following policy: “If the copayment is increased, households employing migrant care workers will be able to access more long-term care services (including benefit cap and items)”? (If not, please explain your reasons)

(1) □Support, which items do you wish to access:

(2) □No opinion (3) □Do not support, reason:

1. Next, I will list several ways to improve LTC 2.0. Please state whether these improvements will encourage you or the care recipient to use these services **(Select all that apply)**

(1) □Ability to flexibly use various types of care services within the current LTC 2.0 benefit cap

(2) □Ability of clients to freely choose the long-term care service institutions or care attendants

(3) □Simplification of application process

(4) □Hospitals to assist in bridging with long-term care services after discharge

(5) □Home visits by professionals to instruct migrant care workers in care skills

(6) □Reducing the costs of using long-term care services

(7) □Provision of nighttime home care services

(8) □Distribution of small grants/care vouchers allowing households to choose LTC 2.0 services

(9) □Other,

1. In what ways do you think the working conditions of migrant live-in care workers should be improved? **(Open-ended response; the interviewer should encourage the interviewee to state at least one method)**
2. In your opinion, how can we effectively improve the emphasis of employers on the rights and interests of migrant live-in care workers? **(Open-ended response; the interviewer should encourage the interviewee to state at least one method)**
3. If information related to long-term care can be consolidated in a mobile app, from a user’s perspective, what information do you think should be included? **(Select the three most important items)**

(1) □Application process for LTC 2.0

(2) □Currently available benefit cap for long-term care services

(3) □Nearby long-term care stations

(4) □Care skills and health education information

(5) □Regulations related to employing migrant care workers

(6) □Other,

Part IV　Basic Personal Information

1. In which county or city does the care recipient currently live **(Can be inputted from database)**
2. What is the gender of the care recipient? **(Can be inputted from database)**

(1) □Male (2) □Female

1. What is the age of the care recipient? **(Can be inputted from database)**

(1) □39 years and below (2) □60–69 years (3) □70–79 years (4) □80–84 years

(5) □85 years and above

1. What is the marital status of the care recipient?

(1) □Married (cohabiting) (2) □Divorced (3) □Widowed (4) □Separated (5) □Never married

1. What is the benefits eligibility of the care recipient? **(Select all that apply)**

(1) □Does not receive social welfare subsidies (2) □Low-income household (3)□Middle-low-income household (4) □Physical or mental disability (5) □Veteran and dependents (6) □Indigenous people

1. What is the education level of the care recipient?

(1) □Illiterate (2) □Home-schooled (3) □Graduated/attended elementary school

(4) □Graduated/attended junior high school (5) □Graduated/attended senior high school (6) □Junior college/university and above

1. What language does the care recipient usually use?

(1) □Taiwanese Hokkien (2) □Hakka (3) □Mandarin (4) □Indigenous languages

(5) □Other

1. What is the living situation of the care recipient?

(D8.1) Living alone (only living with migrant live-in care worker)

(1) □Yes **(skip to QD9)** (2) □No **(continue to QD8.2)**

(D8.2) Do you live with the care recipient?

(1) □Yes **(continue to QD8.3)** (2) □No **(continue to QD8.3)**

(D8.3) Who else lives with the care recipient? **(Select all that apply)**

(1) □Spouse (cohabitant) (2) □Son (3) □Daughter (4) □Other relatives or friends

(5) □None

1. What is your (the employer’s) monthly household income?

(1) □No income (2) □ NT$ 20,000 and below (3) □NT$ 20,001–40,000

(4) □NT$ 40,001–60,000 (5) □NT$ 60,001–80,000　 (6) □NT$ 80,001–100,000

(7) □NT$ 100,001–150,000 (8) □NT$ 150,001–200,000 (9) □NT$ 200,001 and above
