# Supplementary material for: Information sources as determinants of use of formal long-term care: a cross-sectional study in Taiwan
Source: BMC Health Serv Res. 2025 Jul 3;25:910. doi: 10.1186/s12913-025-12814-6 (PMC12225371; doi:10.1186/s12913-025-12814-6)
Supplement: Supplementary file 2 — Supplementary Material 2. [file 12913_2025_12814_MOESM2_ESM.docx]

# Pilot Survey Contact Outcomes

The pilot survey was conducted from June 1 to June 3, 2022, using computer-assisted telephone interviewing (CATI). Calls were primarily made between 6:00 p.m. and 9:00 p.m. to improve contact rates. A total of 275 individuals were contacted (165 in the LTC-user group and 110 in the non-user group), with 3,082 calls placed overall. The figure below illustrates the distribution of contact outcomes, combining both lists.


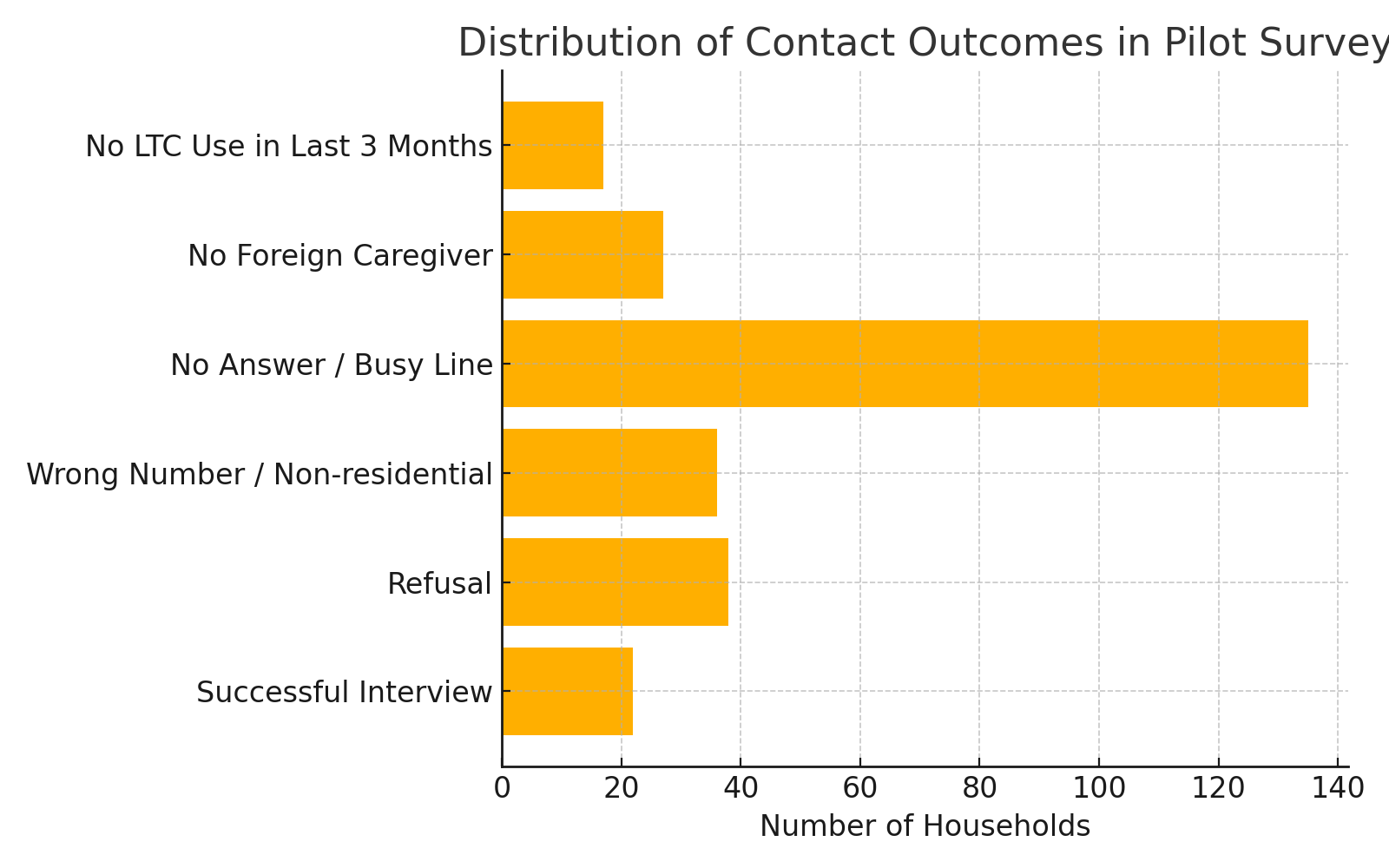


Prior to the main data collection, a pilot test was conducted to assess the feasibility of the telephone survey process. The outcomes of the pilot contact attempts, including interview success, refusal, ineligible numbers, and other call statuses, are summarized in Table 1. Among the 275 contacted individuals, the overall success rate of completed interviews was 8.0%. To evaluate whether the likelihood of successful interviews differed by LTC service use, we compared the distribution of contact outcomes between LTC users and non-users (Table 2). The success rate was 6.7% among LTC users and 10.0% among non-users. A chi-square test revealed no statistically significant difference between groups in interview completion rates (χ² = 2.996, p = 0.318). This suggests that response outcomes were not systematically associated with LTC service use status, and thus unlikely to introduce selection bias in the final sample.

Table1: Distribution of Telephone Contact Results by LTC User Status (Pretest Phase)

| Contact Outcome | LTC-user | | non-user | | Total | |
| --- | --- | --- | --- | --- | --- | --- |
|  | n | % | n | % | n | % |
| Successful Interview | 11 | 6.7 | 11 | 10.0 | 22 | 8.0 |
| Refusal | 21 | 12.7 | 17 | 15.5 | 38 | 13.8 |
| Wrong Number / Non-residential | 21 | 12.7 | 15 | 13.6 | 36 | 13.1 |
| No Answer / Busy Line | 79 | 47.9 | 56 | 50.9 | 135 | 49.1 |
| No Foreign Caregiver | 16 | 9.7 | 11 | 10.0 | 27 | 9.8 |
| Misclassification | 17 | 10.3 | 0 | 0.0 | 17 | 6.2 |
| Sum | **165** |  | **110** |  | **275** |  |

Table2 : Comparison of Interview Completion Rates Between LTC Users and Non-Users

| Contact Outcome | | LTC-user | | non-user | | Total | |
| --- | --- | --- | --- | --- | --- | --- | --- |
|  |  | n | % | n | % | n | % |
| Successful Interview | Yes | 11 | 6.7 | 11 | 10.0 | 22 | 8.0 |
|  | No | 154 | 97.3 | 99 | 90.0 |  | 92.0 |
| Sum | | **165** |  | **110** |  | **275** |  |

Note. A chi-square test was conducted to compare interview completion rates between LTC users and non-users. The difference was not statistically significant (χ² = 2.996, p = 0.318), indicating that completion rates did not differ by LTC service use status.
