# Supplementary material for: Information sources as determinants of use of formal long-term care: a cross-sectional study in Taiwan
Source: BMC Health Serv Res. 2025 Jul 3;25:910. doi: 10.1186/s12913-025-12814-6 (PMC12225371; doi:10.1186/s12913-025-12814-6)
Supplement: Supplementary file 3 — Supplementary Material 3. [file 12913_2025_12814_MOESM3_ESM.docx]

**Sensitivity Analysis: Including LTC Care Workers as a Formal Source**

In the classification of formal information sources (FS), we intentionally excluded “LTC care workers” (referred to as “care attendants” in the original manuscript) due to concerns about potential reverse causality. As LTC care workers are often part of LTC service provision, individuals who already use LTC services are more likely to receive information from them. Including this source could therefore bias the analysis, as it may reflect ongoing service use rather than prior exposure to information. To evaluate whether this exclusion would introduce bias, we conducted a sensitivity analysis by redefining FS to include “LTC care workers,” hereafter referred to as FS(c).

Table 1 presents the distribution of the number of formal information sources selected under both definitions. The proportion of respondents who reported at least one FS source changed only slightly, from 63.9% under the original FS definition to 66.7% under FS(c).

Table 2 further displays the results of logistic regression models examining the association between information source types and LTC service use. In the model 1, which used the original FS definition, the adjusted odds ratio (OR) for FS was 1.470 (95% CI: 1.10–1.96). In Model 2, which included LTC care workers in the FS definition, the OR remained consistent at 1.468 (95% CI: 1.13–1.90). The odds ratios for informal sources (IS) were also stable across both models, at 0.666 (95% CI: 0.54–0.82) in Model 1 and 0.650 (95% CI: 0.53–0.80) in Model 2.These consistent results indicate that including or excluding LTC care workers as a formal information source did not materially affect the observed association between information source types and LTC service use. Thus, our decision to exclude this source in the primary model is methodologically sound and seems unlikely to introduce bias.

Table 1. Distribution of the Number of Formal Information Sources on LTC Services, With and Without Inclusion of LTC care workers.

| No. of  formal sources | FS | | FS(c) | |
| --- | --- | --- | --- | --- |
|  | N | % | n | % |
| 0 | 159 | 36.1 | 147 | 33.3 |
| 1 | 214 | 48.5 | 212 | 48.1 |
| 2 | 52 | 11.8 | 62 | 14.1 |
| 3 | 12 | 2.7 | 10 | 2.3 |
| 4 | 4 | 0.9 | 6 | 1.4 |
| 5 | 0 | 0 | 4 | 0.9 |
| Total | 441 | 100 | 441 | 100 |

NOTE. FS indicates the number of formal information sources from which respondents reported learning about LTC services, excluding LTC care workers. FS(c) includes LTC care workers as an additional formal source.

Table 2. The effects of numbers of information sources on use of LTC services, with and without inclusion of LTC care workers as formal sources.

|  | Odds ratios (95% CI) | |
| --- | --- | --- |
|  | Model 1 | Model 2 |
| FS | 1.470 (1.10-1.96)^**^ | 1.468 (1.13-1.90)^**^ |
| IS | 0.666 (0.54-0.82)^***^ | 0.650 (0.53-0.80)^***^ |

NOTE. 1. Both Model 1 and Model 2 were adjusted for multiple covariates based on employer and care recipient characteristics. These included the employer’s gender, age, and education level, as well as their caregiving role. For care recipients, both models adjusted for gender, age, and education level, along with their living arrangement. The model also included the total number of caregiving tasks needed and the number of tasks requiring assistance, both categorized into four levels. Additional covariates were the duration of employing a migrant caregiver, monthly household income, household financial status under the Public Assistance Act, and residential region.

2. ^*^*p* < 0.05. ^**^*p* < 0.01. ^***^*p* < 0.001.
